# Measuring competency-relevant knowledge in the competency-oriented student progress test

**DOI:** 10.3205/zma001299

**Published:** 2020-02-17

**Authors:** Andreas Möltner, Stefan Wagener, Mirka Burkert

**Affiliations:** 1 Medical Faculty of Heidelberg, Baden-Württemberg Center of Excellence for Assessment in Medicine, Heidelberg, Germany

**Keywords:** progress test, reliability, discriminant validity

## Abstract

**Background: **Since 2013 a competency-oriented student progress test (SKPT) has been administered at a number of German medical schools. The questions are generated on the basis of a two-dimensional blueprint, on which one axis contains the five competency domains – communicative competence (CO), practical clinical competence (CP), theoretical clinical competence (CT), scientific competence (SC), and professional decision-making competence (PR) – that form part of the competency model of the National Competency-based Catalogue of Learning Objectives for Undergraduate Medicine (NKLM). The feedback for students is structured in part according to these domains. The aim of our study is to examine if

1. the results differentiated by competency domain show adequate measurement accuracy and
2. if the results for the different domains also contain different information.

**Methods: **The SKPTs for the years 2013 to 2017, taken by a total of 3,027 students, were examined. The measurement accuracy was determined using the coefficient glb (greatest bound to reliability) and the standard error of measurement; discriminant analysis of the principal components was carried out to demonstrate differentiation between the competency domains.

**Results: **The reliability of the competency domains was above 0.8 for all SKPTs; exceptions to this were seen in two of the tests for CO and PR that had a reliability of 0.7–0.8. The results for all of the individual competency domains differed in their informational content compared to the overall of the other domains; the same applies for all pairwise comparisons, with the exceptions of CP and CT.

**Discussion:** The SKPT feedback for students that is differentiated by competency domains basically fulfills the requirements for measurement reliability and distinctness. An improvement of the measurement quality for CO and PR and a better differentiation between CP and CT is desirable.

## Zusammenfassung

**Hintergrund: **Seit dem Jahr 2013 wird an einer Reihe medizinischer Fakultäten ein studentischer kompetenzorientierter Progresstest (SKPT) durchgeführt. Die Erstellung der Fragen erfolgt auf Basis eines zweidimensionalen Blueprints, dessen eine Achse aus den auf dem Kompetenzmodell des NKLM basierenden fünf Kompetenzbereichen „Kommunikative Kompetenz“ (KO), „Klinisch-praktische Kompetenz“ (KP) „Klinisch-theoretische Kompetenz“ (KT), „Wissenschaftskompetenz“ (WI) und „Professionelle Handlungskompetenz“ (PH) besteht. Die Rückmeldung an die teilnehmenden Studierenden erfolgt u. a. differenziert nach diesen Bereichen. Ziel der Studie ist, zu prüfen, ob

1. die nach Kompetenzbereichen differenzierten Ergebnisse eine hinreichende Messgenauigkeit aufweisen und
2. ob die Ergebnisse zu verschiedenen Bereichen auch unterschiedliche Informationen enthalten.

**Methoden: **Untersucht wurden die SKPTs der Jahre 2013 bis 2017 mit zusammen 3027 Teilnehmern. Die Messgenauigkeit wurde mit dem Koeffizient glb und dem Standardmessfehler bestimmt, zum Nachweis der Differenziertheit der Kompetenzbereiche wurde eine Diskriminanzanalyse der Hauptkomponenten eingesetzt.

**Ergebnisse: **Die Reliabilität der Kompetenzbereiche war in allen Progresstests über 0.8, Ausnahmen hiervon waren in zwei der Tests KO und PH mit einer Reliabilität von 0.7–0.8. Die Ergebnisse aller Bereiche unterschieden sich hinsichtlich ihres Informationsgehalts jeweils vom Gesamt der anderen Bereiche, gleiches gilt mit Ausnahme von KP und KT für sämtliche paarweisen Vergleiche.

**Diskussion: **Die nach Kompetenzbereichen differenzierte Rückmeldung der Leistungen im SKPT erfüllt im Wesentlichen die Anforderungen an Messzuverlässigkeit und Eigenständigkeit. Eine Verbesserung der Messgüte bei den Bereichen KO und PH sowie eine bessere Differenzierung der Bereiche KP und KT ist wünschenswert.

## 1. Background

Competency-based medical education (CBME) has received special attention over the past 20 years in connection with curriculum development at medical schools and health policy [3], [26]. As a consequence, this must also be reflected in educational assessments [5]. This has led to a veritable flood of publications on competency-based testing (see the critical discussion of this in [11]), with a particular focus on practical and workplace-based methods to assess communication skills, professional competence, and so on. Less attention has been paid to assessments using traditional multiple-choice questions (MCQs) since, assuming that competencies are primarily action-based, less importance is placed on the simple measurement of knowledge. Nonetheless, knowledge is an essential prerequisite for taking competent action and can be measured using competency-based tests of knowledge [23]. MCQs continue to offer substantial advantages to accomplish this. Due to the generally brief amount of time needed to answer a MCQ, it is possible to ask a large number of questions on a test that then, relative to the time needed, allows for distinctly better coverage and representation of the curricular content that is being tested than do open-ended essay formats, which are laborious to grade, or practical assessment formats. In addition, objectivity and a high reliability are easier to achieve than, for instance, with workplace-based assessments in which there is the risk that their seemingly higher validity is rendered void by insufficient standardization.

As part of the BMBF-funded project *Medical Education Research – Lehrforschung im Netz BW* (MERLIN, http://www.merlin-bw.de), a competency-oriented student progress test (SKPT) was designed in 2013 by the Baden-Württemberg Center of Excellence for Assessment in Medicine (Kompetenzzentrum für Prüfungen in der Medizin Baden-Württemberg).

Progress tests are recognized and used in medical education to map learning progress over the course of medical study [28]. To do this, students of all semester levels (year of study) are given the same test. If there is sufficient equivalence (same difficulty level) between the tests administered in consecutive years, students are able to see their progress over the course of their studies and receive corresponding feedback. Progress tests are administered at different medical schools in Germany [18]. In general, these tests have two main functions: a) to give students ongoing feedback on their individual academic proficiency and b) to give medical schools the opportunity to monitor curricula, observe academic progress in different student cohorts and compare different curricula [24], [34].

The questions on the SKPT derive from a two-dimensional blueprint whose axes contain the eight subjects identified by the German medical licensing regulations (ÄAppO) and the individual competencies that are grouped into five competency domains in the National Competency-based Catalogue of Learning Objectives for Undergraduate Medical Education (NKLM) [4]. This blueprint was created by three inter-university and interdisciplinary expert groups who were tasked with grouping the domains in the NKLM in clusters referred to as “competency domains” and creating “subject groups” out of the subjects listed in the medical licensing regulations. These groups needed to reflect the best balance possible between preclinical and clinical subjects (see table 1 (Tab. 1) and table 2 (Tab. 2)).

A special aspect of the SKPT is that the questions are generated by students who are trained during multiple annual workshops. These students compose questions based on the competencies defined in the NKLM with reference to the subject groups in order to fill in the cells of the blueprint in table 2 (Tab. 2). The number of existing questions per cell is documented in an ongoing manner so that, especially near the end of the process, only the questions still needed to fill the empty cells need to be formulated. See [33] for a detailed description.

The progress test is administered each year in November/December (as of 2015 in cooperation with the Institut für Kommunikations- und Prüfungsforschung gGmbH). All medical students enrolled at universities where the SKPT is offered can take the test. In 2017 the SKPT was administered at a total of 16 medical schools: Dresden, Erlangen-Nürnberg, Freiburg, Gießen, Hannover, Heidelberg, Homburg, Krems (Austria), Leipzig, Magdeburg, Mannheim, Marburg, LMU München, Tübingen, Ulm, and Witten/Herdecke. Participation is voluntary, except at the private Karl Landsteiner University in Krems, Austria, where participation is mandatory.

The test consists of 120 Type A MCQs (one correct response out of four to five possible responses) plus the additional option of “I don’t know” and ten situational judgment test questions (SJT) to measure social competencies [20]. An exception to this was the first progress test in 2013 that consisted of 144 Type A questions and no SJT.

The “I don’t know” option is frequently used in progress tests and other formative tests to enable test-takers to explicitly document their knowledge deficits and to avoid any guessing based on the possible response options [13], [22].

### Example question from the Theoretical Clinical Competency domain (CT) on the 2017 SKPT

You are treating a 12-year-old child with pneumonia on the pediatric ward. The patient has a history of frequent respiratory complaints and infections. During an ultrasound examination you determine situs inversus, meaning that the organs are reversed from their normal positions. As part of a bronchoscopy you then send a biopsy for histological analysis.

Which diagnosis are you expecting based on the histology?

1. Defect in the cell-to-cell junctions
2. Defect in the kinocilia
3. Formation of pseudostratified epithelium
4. Absence of surface differentiations
5. Defect in the basement membrane
6. I don’t know

The correct answer is B. Additional examples are included in the attachment 1 . All of the SKPT questions and their explanations can be found on the publicly accessible webpage https://www.komp-pt.de/fragen-aus-dem-progresstest/.

After taking the test all examinees receive feedback on their performance (number of points earned) which is differentiated according the subject groups and competency domains. This feedback is absolute (criteria-based), relative in comparison to the other participants at the same semester level (standards-based), and – if prior tests have been taken – longitudinal to show the gain in knowledge (progress) compared to earlier scores (ipsative).

The aim of this study is to ascertain

1. if the scores differentiated for each competency domain have sufficient measurement precision and
2. if the questions of the different competency domains constitute empirically distinct clusters.

Both of these issues are especially important for the usefulness of the feedback given to students:

1. feedback is only beneficial if the graded performance has been reliably measured; and
2. differentiated feedback is only meaningful if the individual scores reflect different content and thus are not redundant.

In the terminology used by Campbell and Fiske [1], the second issue involves proving “discriminant validity” (also referred to as “discriminative validity”). The term “validity” has been the subject of intense discussion over the past 30 years. Many authors critically view the use of different terms to describe validity, e.g. predictive, convergent, discriminant; (a thorough description of this can be found in [17]). In the present study we use this term to refer to its “classic” definition: the questions assigned to competency domains form “scales” which measure the performance of different tasks (see the discussion in [6]). These “scales” are also supposed to be reflected in the responses of the progress test-takers. Basically, questions grouped together in the same domain should be answered similarly well (or badly).

## 2. Methods

### 2.1. Data collection

The SKPT was administered once annually between 2013 and 2017. In the first two years the SKPT was taken as a paper-and-pencil test at each participating medical school; as of 2015 it is available online.

Test announcement, student registration and conducting the progress test at each university are handled individually by each medical school. The overarching coordination is in the hands of the Baden-Württemberg Center of Excellence for Assessment in Medicine (Kompetenzzentrum für Prüfungen in der Medizin Baden-Württemberg), which is housed at the Medical Faculty of Heidelberg. For more details on the administration of the test, please see [33], [29], [30], [31], [32].

The SKPT is designed as a formative test that can be taken voluntarily. However, at the medical school in Krems, Austria, the SKPT is mandatory. Since it has been shown in various analyses (not presented here) that the Krems student group differs clearly from the voluntary participants at the other medical schools, the Austrian group has been excluded from the following analysis. A second exclusion criterion was the number of answered questions. The present study includes only those test-takers who answered at least 100 of the 120 questions, meaning that they marked one of the four or five possible answers or indicated that they did not know (see table 3 (Tab. 3)). Only complete datasets have been included for the first two paper-based SKPTs from 2013 and 2014. Table 4 (Tab. 4) shows the number of participants in the analysis by year of study. The ten SJT questions from 2014 through 2017 did not fall within the scope of this study.

Prior to taking the SKPT, the students consented to the use of their pseudonymized data for the purpose of quality assurance and academic research.

#### 2.2. Statistical analyses

##### 2.2.1. Evaluation of the questions

The questions that were generated based on the blueprint were type-A MCQs with the additional option to respond with “I don’t know.” One point is assigned if the correct answer is chosen. For this analysis, incorrect responses, “I don’t know” and questions left blank were treated the same and assigned zero points (see [22] for alternative grading approaches in which “I don’t know” and incorrect responses are treated differently). After test administration, there was a second review (post-review) of the questions, which was based on student comments and the statistical analysis. If it was determined that more than one response was possible for a question, the test-takers received one point if they had selected one of the correct answers. Based on the post-reviews, we had to exclude between three to eight questions on the SKPTs for the years 2013 to 2017 for being flawed (see table 3 (Tab. 3)).

##### 2.2.2. Reliability and measurement precision

The greatest lower bound of reliability (glb) is used to estimate the reliability of the competency domains [7], [25]. This is also the optimal algebraic estimate of the reliability for non-homogenous scales (in this case, Cronbach’s α, as a measure of the internal consistency, yields an underestimation of reliability).

Reliability is a relative measure of measurement precision based on the participant population; the standard error of measurement serves as an absolute measure of measurement precision and is calculated from the reliability and the standard deviation of the scale values for the examinees [9].

When comparing students who are at the same semester level, reliability is relevant also as it refers to these particular subpopulations. In these cases, lower values are to be expected in comparison to the overall reliability since, assuming almost the same measurement error, the variance in the number of correctly answered questions among examinees at the same academic level is lower than the number for all examinees across all semester levels (see [33]).

##### 2.2.3. Delineating the competency domains (“discriminant validity”)

Testing whether or not the different competency domains denote empirical differences becomes somewhat complex given the progress test’s design: the individual questions are not only assigned to one competency domain, but also to a subject or a subject group. As a consequence of this design, the constructs defined by the two axes of the blueprint already overlap each other (for a detailed explanation of construct overlap, see [35], among others). For this reason, we cannot assume that the competency domains are directly apparent in the data when applying methods of factor or cluster analysis (compare the factor analysis of the validity and reliability of competency constructs in [12]).

Therefore, we chose Fisher’s linear discriminant analysis as the methodical approach. The objects in this case are the individual questions with the competency domain as the grouping variable. The number of points scored by the test-takers for the questions (response patterns) are the predictors. One problem here is that more people have participated than there are questions. Analogous to the approach taken for a principal component regression, data reduction was therefore carried out by determining the principal components. Linear discriminant analyses were then carried out with a reduced number of principal components. This “discriminant analysis of the principal components” (DAPC) is used for similar reasons in genetic analyses in which the number of predictors exceeds the number of objects to be classified ([8], see also note 2 below).

The first analytical step (determining the principal components) does not involve any distributional assumptions. The extraction of the principal components serves to reduce the original data to a few components in the response patterns which best approximate the data according to the least squares criterion. Fisher’s linear discriminant analysis is a special case of linear discriminant analysis in which the a priori group sizes are assumed to be equal. In this special case, no normal distributional assumption is made for the analysis. Calculation of the p-values for the group comparisons (see below) is done using t-tests that in principle are based on normal distributional assumptions but are known to be robust to violations of these assumptions (we also performed a non-parametric test by means of a randomizing test whose results are substantially identical to the t-test presented here and are therefore not reported here for reasons of clarity).

One question associated with the method of factor analysis and one that generally cannot be answered satisfactorily is the question concerning the determination of the number of principal components to be used. For the different SKPT, no consistent value over the years has been yielded by scree plots [21] or from Onatski’s method [19], which is why we extracted ten principal components, meaning twice as many components as competency domains (the following results are less sensitive in relation to the number of the extracted components; analyses with more than six components yield nearly identical results).

To answer the question if the individual competency domains are different from the entirety of all other domains, a one-against-the-rest analysis and a pairwise classification of all competency domains against each other (one-against-one) were carried out [10], [27]. Tests of significance were done in each case by combining the individual p-values from the five progress tests for the years 2013 to 2017 using Fisher’s method (Fisher’s combined probability test). Significance was defined as α= 0.05.

## 3. Results

### 3.1. Reliability and measurement precision

**Figure 1:**
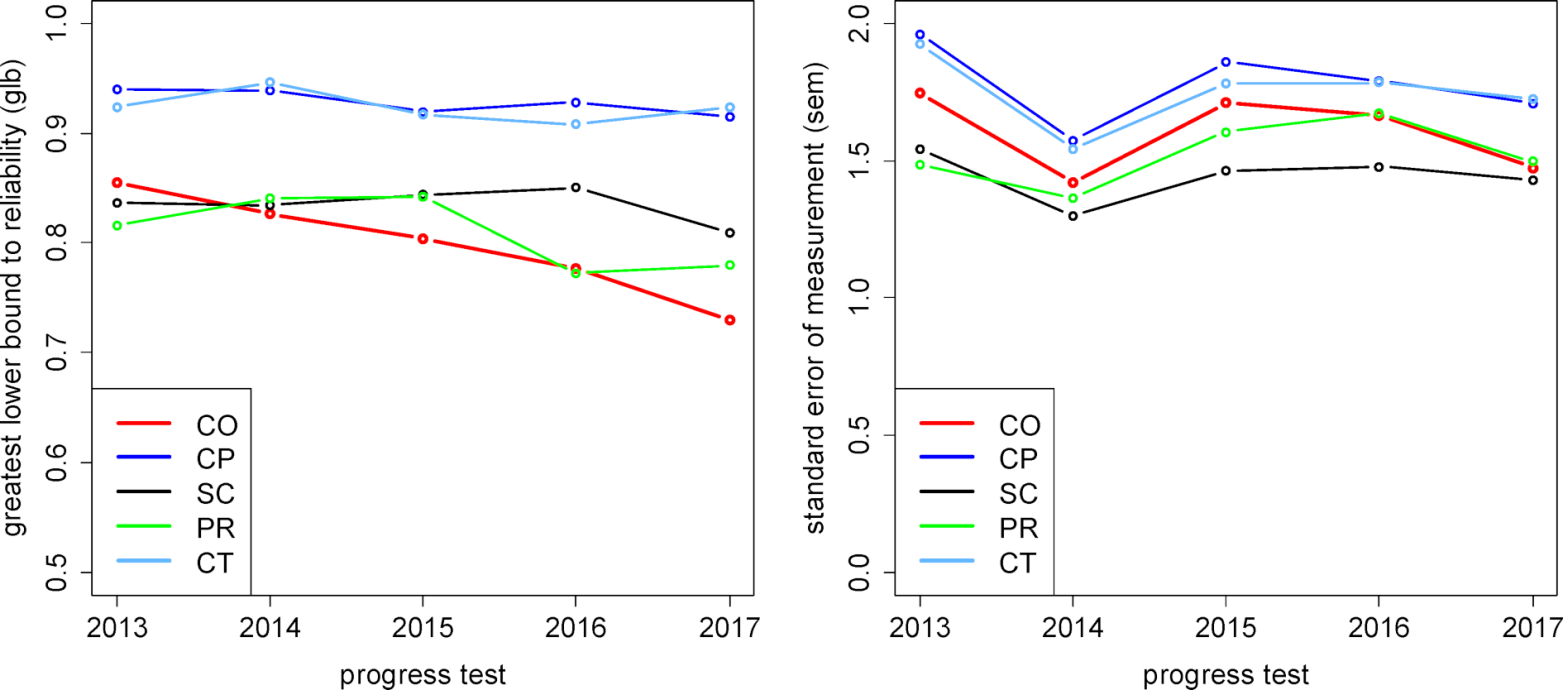
Reliability glb (left) and standard error of measurement sem (right) for the competency domains in the progress tests 2013–2017.

The number of questions, reliability coefficients glb, and the corresponding standard error of measurements (sem) are listed in table 5 (Tab. 5); a visual depiction of glb and sem over the years is presented in figure 1 (Fig. 1).

Practical clinical competence (CP) and theoretical clinical competence (CT) are measured as nearly stable with a high reliability (more than 0.90); (one notes that the number of the questions in these two competency domains is higher than in the other competency domains, see the blueprint). Scientific competence (SC) is likewise stable over time with a reliability of around 0.80-0.85. For professional decision-making competence (PR) and communicative competence (CO), a decrease to a reliability below 0.80 is seen for the last two years, most distinctly for communicative competence (CO) with a reliability of 0.73 for the 2017 progress test (see figure 1 (Fig. 1), left diagram).

For the standard error of measurement (see figure 1 (Fig. 1), right diagram), a distinct reduction in 2014 is visible that can be traced to the fewer number of questions in comparison to the SKPT for 2013. Afterward, the standard error of measurements for all competency domains remain virtually the same since 2015. Here, too, varying numbers of questions in the competency domains must be taken into account; longer scales also have a larger absolute sem. This is also reflected in figure 1 (Fig. 1) (left): practical clinical competence (CP) and theoretical clinical competence (CT) are the longest scales (with 30 questions each on the blueprint), and scientific competence (SC) has the shortest scale with only 18 questions.

**Figure 2:**
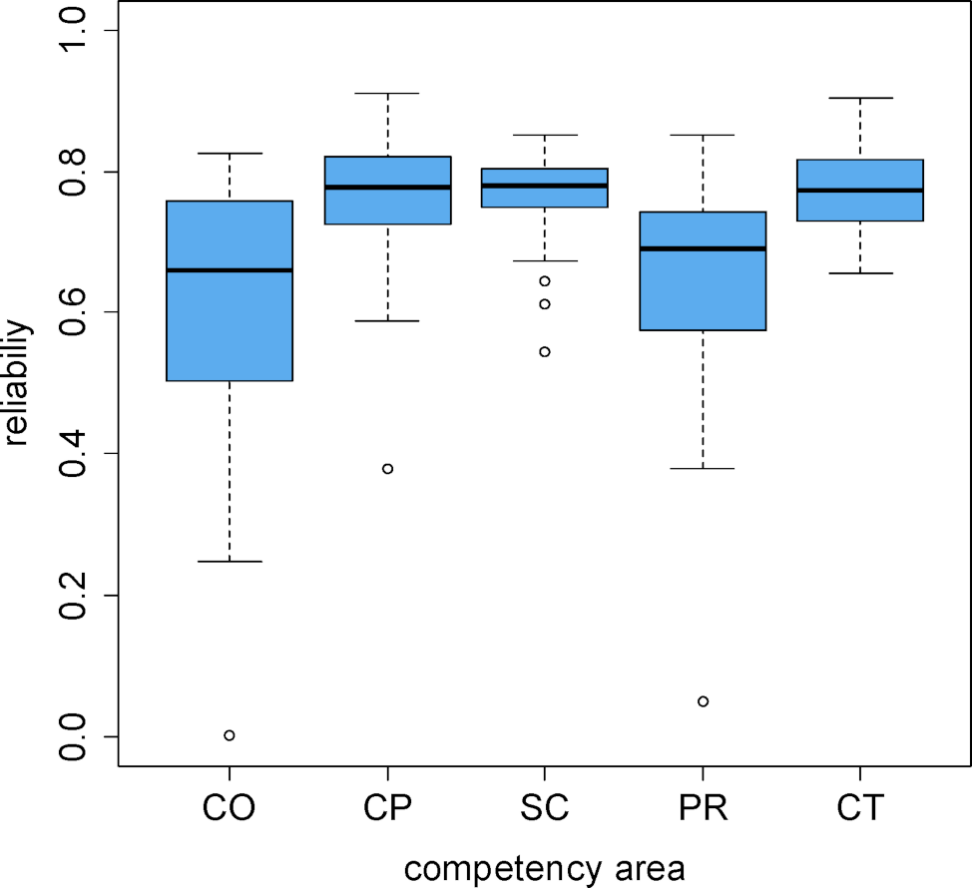
Reliabilities within year of study for the individual competency domains. Each box represents the 30 individual reliabilities of the five progress tests and six levels of study reflected in years of study.

The median values for the reliabilities in the subpopulations at the same semester level are presented in table 6 (Tab. 6) according to both year of study and competency domain. In addition, figure 2 (Fig. 2) shows the distribution of these 30 individual reliabilities for the different competency domains. While the measurement reliabilities for the majority of reliabilities for the practical clinical, theoretical clinical, and scientific competency domains (CP, CT, SC) are above 0.7 (CP 83%, SC 80%, CT 90%), these percentages are distinctly lower for communicative competence (CO) with 37% and for professional decision-making (PR) with 47%. In particular, for these individual SKPTs and semester levels it must be established that there are low reliabilities under 0.4 (see figure 2 (Fig. 2)). Low measurement reliabilities appear to be especially frequent for sixth-year students (percentages of the reliabilities over 0.7 for the first through fifth years of study: CO 44%, CP 84%, SC 84%, PR 56%, CT 96% (see table 6 (Tab. 6)).

#### 3.2. Delineation of the competency domains (“discriminant validity”)

##### 3.2.1. Delineation of each competency domain in relation to all other domains

**Figure 3:**
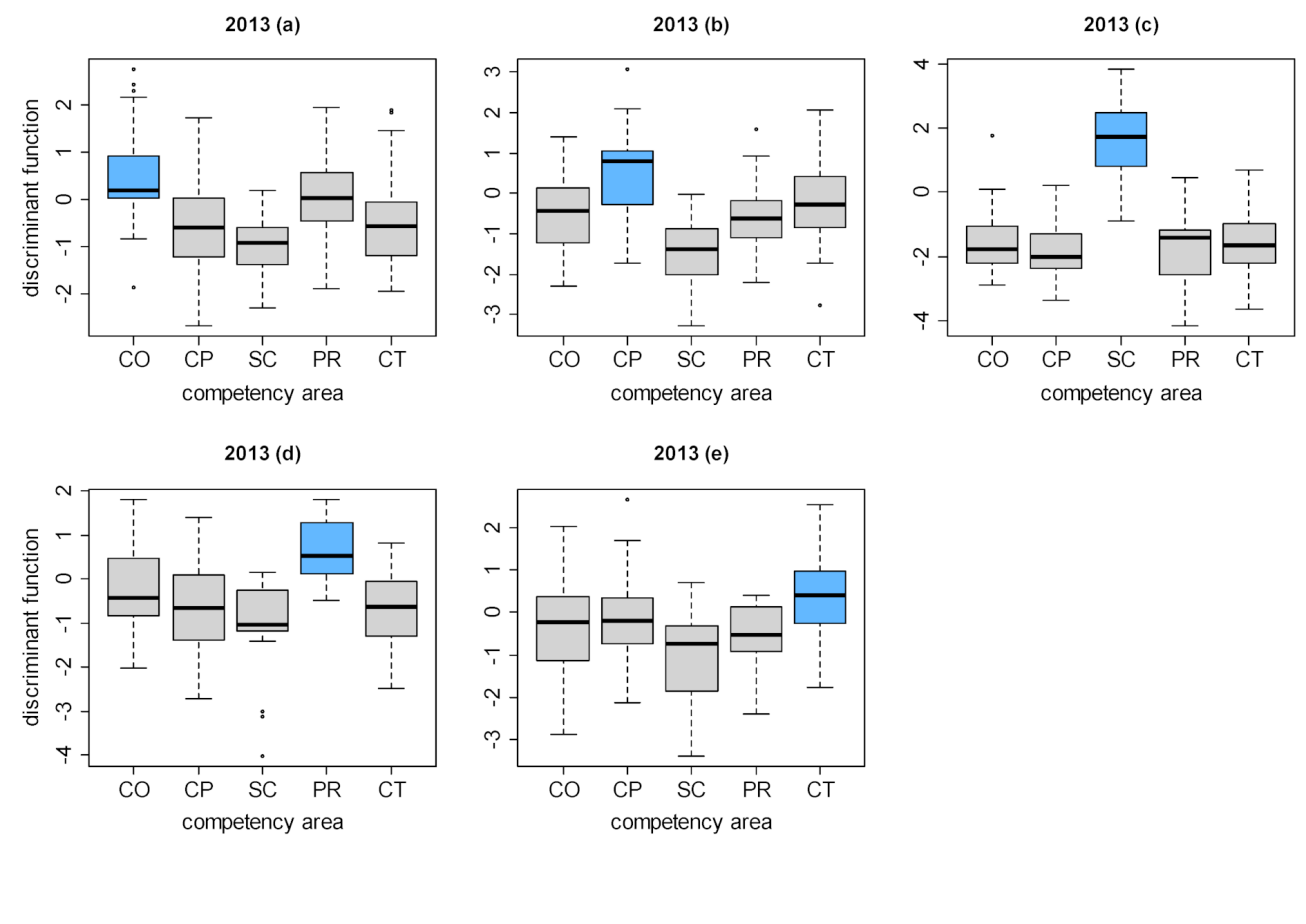
Distributions of the discriminant function values for the questions in the competency domains after performing two-class discriminant analysis for each domain in relation to all other domains (one-against-the-rest) for the 2013 progress test.

**Figure 4:**
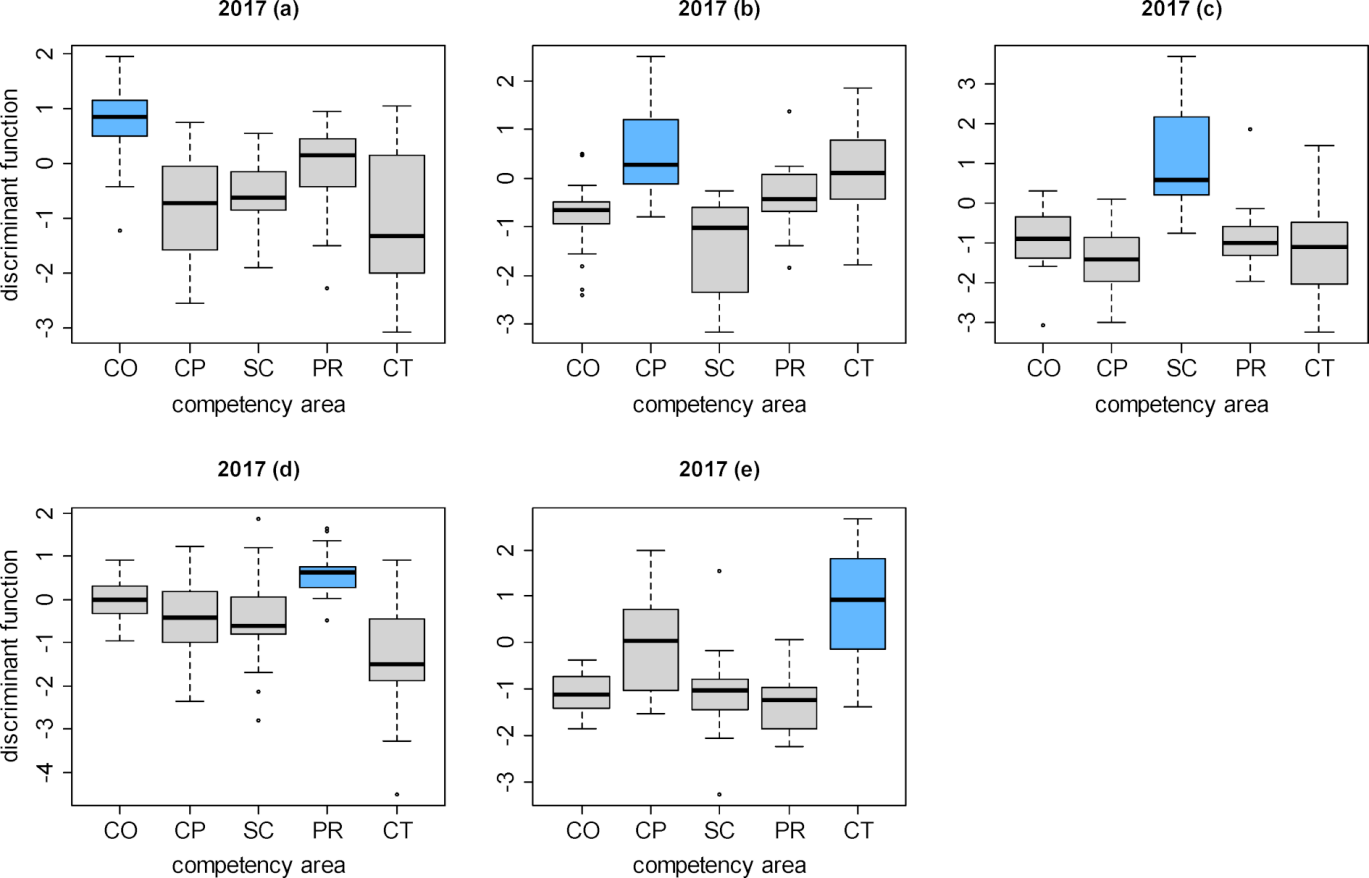
Distributions of the discriminant function values for the questions in the competency domains after performing two-class discriminant analysis for each domain in relation to all other domains (one-against-the-rest) for the 2017 progress test.

To provide examples, the results of the two-class discriminant analyses of a competency domain in relation to every other competency domain are presented in figure 3 (Fig. 3) and figure 4 (Fig. 4) for the progress tests given in 2013 and 2017 (the results for 2014 to 2016 are very similar). On the ordinate axes are the values of the discriminant function of the individual questions in the form of a box plot. Clear overlapping in the box plots of two competency domains points to construct overlapping in the associated questions. When the boxes are clearly separated this indicates that the two groups of questions, and hence the two competency domains, are distinct from each other.

Thus, the questions belonging to scientific competence (SC) in all years form a clearly delineated cluster: it is highly probable that whoever answers one question from this competency domain correctly, also answers the other questions in this domain correctly (see figure 3 (Fig. 3), diagram c and figure 4 (Fig. 4), diagram c). In contrast, there is a high degree of overlapping between practical clinical competence (CP) and theoretical clinical competence (CT) (see figure 3 (Fig. 3), diagrams b, e; and figure 4 (Fig. 4), diagrams b, e).

For inferential statistical testing to see if the individual competency domains differ from the entirety of all other domains, the individual p-values for 2013 through 2017 were combined in Fisher’s probability test (one notes that the individual p-values are not used for significance testing). There are significant values for all five domains (see table 7 (Tab. 7)).

##### 3.2.2. Pairwise delineation of the competency domains

**Figure 5:**
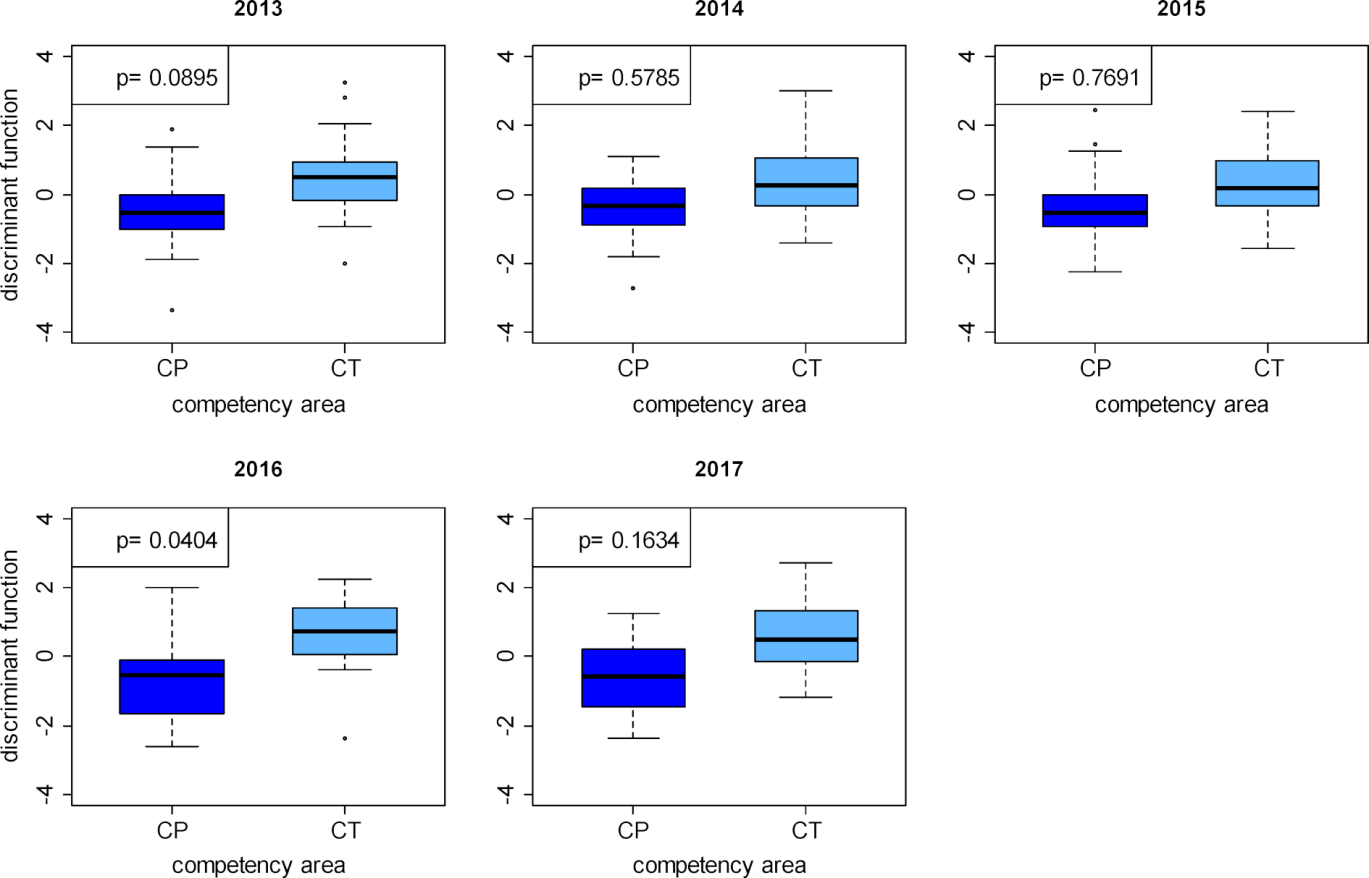
Distributions of the discriminant function values for the questions in the practical clinical competency domain and the theoretical clinical competency domain after performing two-class discriminant analysis for the progress tests 2013–2017.

**Figure 6:**
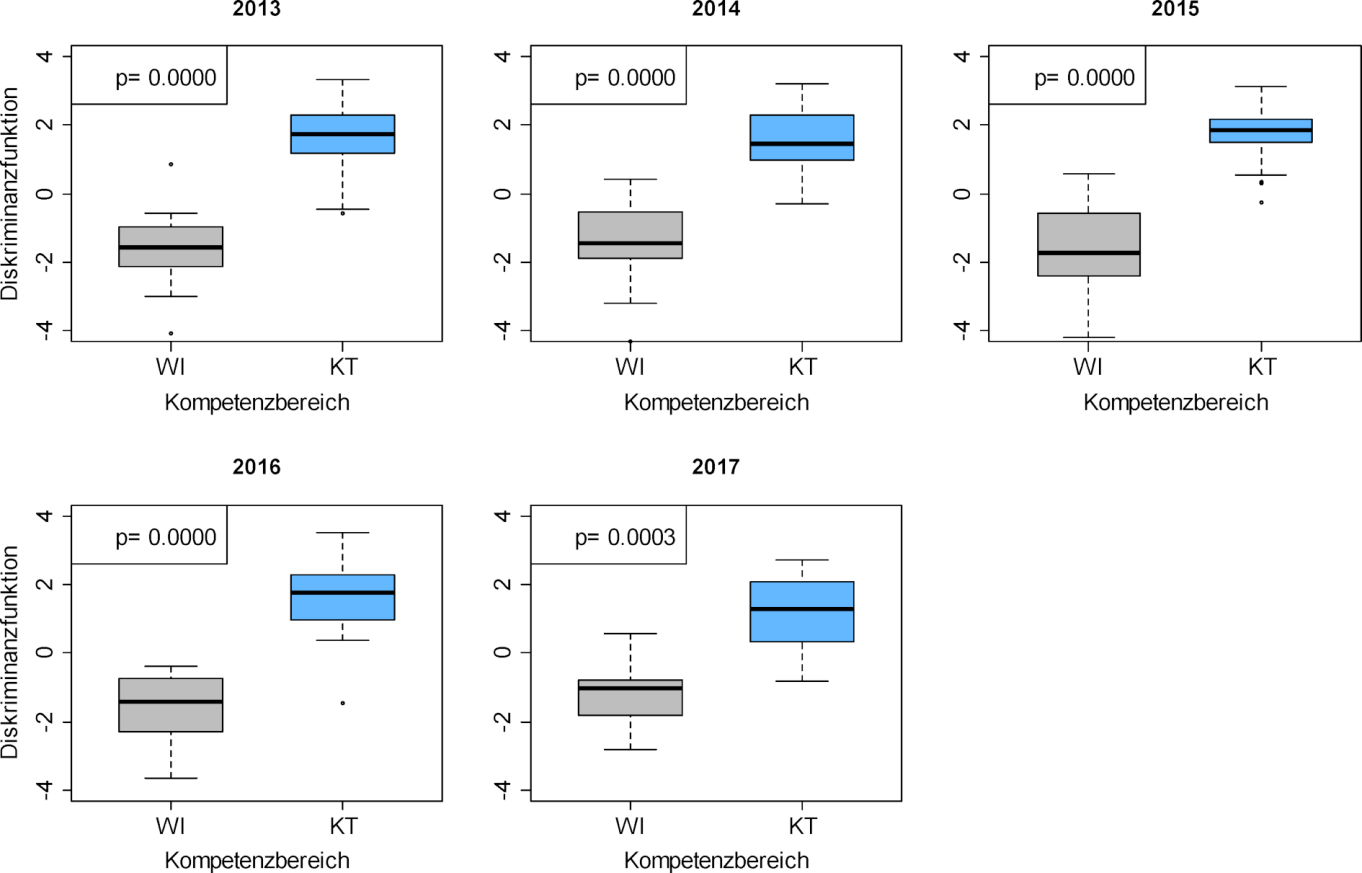
Distributions of the discriminant function values for the questions in the scientific competence domain and the theoretical clinical competence domain after performing two-class discriminant analysis for the progress tests 2013–2017.

To provide examples of the ten possible paired comparisons, we present the values of the discriminant function of the questions for the comparison between practical clinical competence (CP) and theoretical clinical competence (CT) in figure 5 (Fig. 5), and the values comparing scientific competence (SC) and theoretical clinical competence (CT) in figure 6 (Fig. 6).

We present the p-values, combined from the individual values for the different SKPTs, for all paired comparisons in table 8 (Tab. 8). All of these pairwise comparisons are significant, with the exception of the comparison between the practical clinical (CP) and the theoretical clinical (CT) competency domains.

These significant differences remain even after applying the Bonferroni-Holm method to adjust the ten tests: for this, the individual p-values of the tests are ordered in ascending order according to size (p_[1]_≤p_[2]_≤…≤[_10]_). Significant are precisely those p_[k]_ for which the inequalities p_[i]_≤α/(11 – i) are fulfilled for all i≤k. If an inequality is not achieved for any smaller p[i], then no larger p[k] can still be considered significant.

For eight of the tests the result is p<0.001, making these significant based on p_[1]_≤0.05/10, p_[2]_≤0.05/9, … p_[8]_≤0.05/3; in addition, p_[9]_=0.002≤0.05/2=0.025 is also significant. Only the p-value for the comparison between the competency domains CP and CT, which is p_[10]_=0.091, does not fulfill the condition p_[10]_≤0.05/1.

## 4. Summary and discussion

The competency domains of practical clinical competence (CP), theoretical clinical competence (CT) and scientific competence (SC) have been reliably measured by five SKPTs over the years (reliability over 0.80). Not quite so satisfactory are the questions covering the domains of communicative competence (CO) and professional decision-making competence (PR), with reliabilities still over 0.73. The questions for the different competency domains also represent empirically different domains. One exception is seen in the groups of questions covering practical clinical and theoretical clinical competence, between which no delineation can be empirically demonstrated (p=0.091).

With few exceptions, the measurement reliabilities within the student cohorts by year of study show satisfactory values for the practical clinical, theoretical clinical and scientific competency domains (CP, CT, SC). Limitations are seen in the reliabilities of the domains communication and professional decision making (CO, PR), which frequently still do not reach 0.7. However, it must be noted that these domains are covered by only 22 and 20 questions, respectively, numbers that are generally inadequate for achieving a sufficiently high reliability using Type A MCQs even on the well-prepared summative, subject-specific tests given at medical schools.

It is thus shown that on competency-based knowledge tests the generation of questions to measure practically relevant knowledge is also possible with a limited number of questions and – using the terminology of Cronbach and Meehl [2] – represent the different constructs in the blueprint intended for the competency domains.

The exceptions to this are the practical clinical and theoretical clinical competency domains (CP/CT), which, although they differ significantly from the other competency domains, do not differ from each other. Because the discriminant analysis applied here explicitly allows construct overlap, the fact that no delineation can be drawn between these two domains cannot serve as an explanation. The reason for this non-separability could be rules that are too vague to clearly assign a question to only one of the two domains. An alternative explanation could be that within the context of medical expertise these two domains are very strongly connected in terms of content and that this knowledge is largely acquired by students at parallel points in time. Despite the semantic differences of each domain, this would lead to no detection of an empirical difference in the test question responses: whoever has the knowledge to answer the questions in one domain also has the knowledge to answer the questions in the other.

Discriminant analysis of the principal components (DAPC) proved itself to be methodically suitable for empirically tracing the underlying structure of the blueprint axis for the competency domains.

One consequence arising from these results for future progress tests should be an attempt to improve the measurement reliability for competence in communicative (CO) and professional decision-making (PR). This could be achieved by increasing the number of questions for each domain. To avoid enlarging the scope of the SKPT, it would be conceivable to reduce the number of questions asked in the domains of practical clinical competence (CP) and theoretical clinical competence (CT). Delineation of the content of these two domains should also be verified. If empirical discrimination remains impossible to determine, then these two domains could be combined in the feedback given to students.

## Notes

1. Parts of the study concerning the 2013 and 2014 progress tests were reported at the 2014 GMA conference in Hamburg and at RIME 2015 in Munich [14], [15].
2. The authors are unaware of an original source on discriminant analysis of principal components; the earliest mention we found is in a paper on the distribution of larger mammals in a national park in Tanzania [16].

## Funding

This study was undertaken within the scope of the MERLIN II project that is funded by the Federal Ministry of Education and Research (01PL17011C).

## Competing interests

The authors declare that they have no competing interests. 

## Supplementary Material

Examples of competency-specific questions from the 2017 competency-based progress test

## Figures and Tables

**Table 1:**
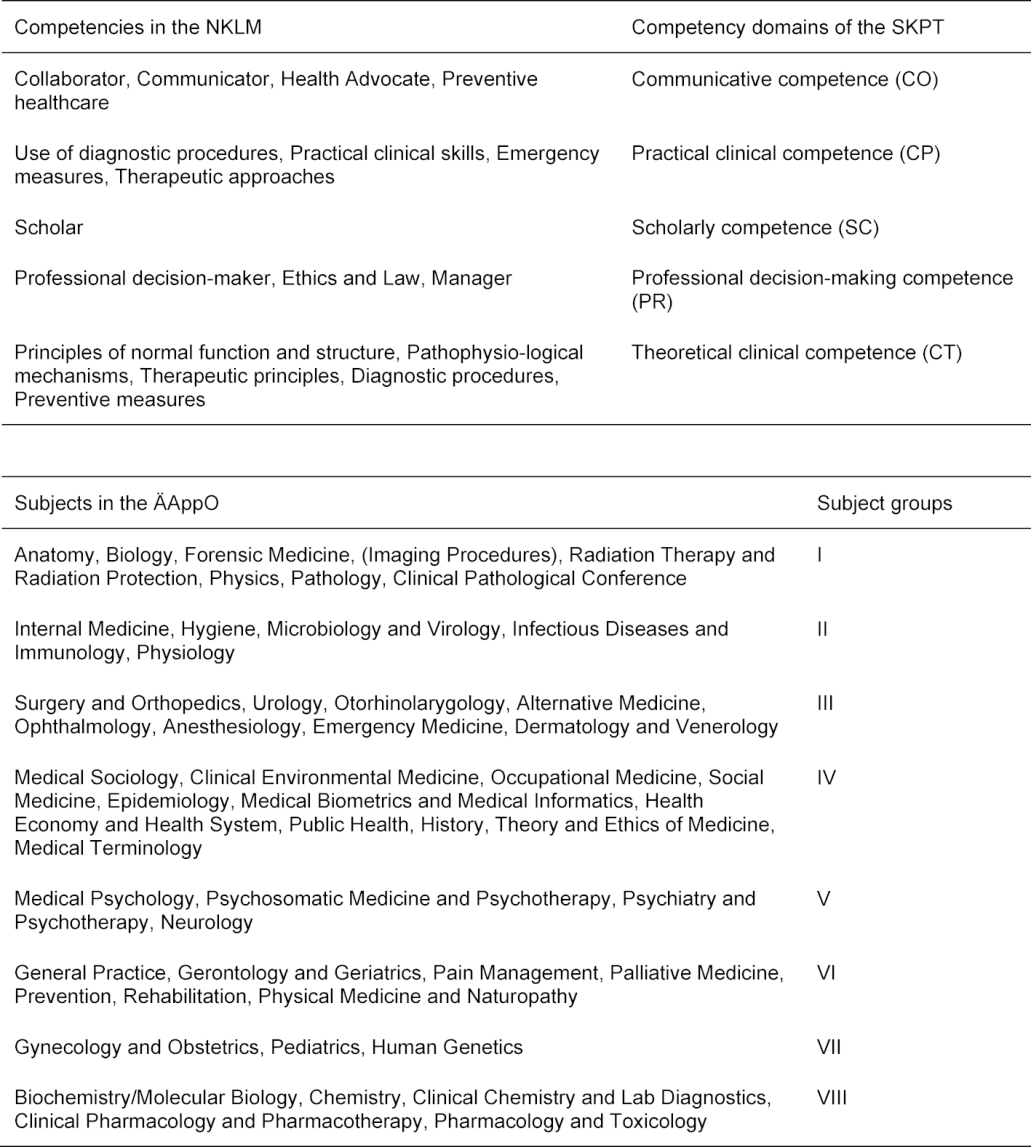
Blueprint axes for the SKPT: assignment of the individual competencies in the National Competency-based Catalogue of Learning Objectives in Undergraduate Medicine (NKLM) to the competency domains of the competency-oriented student progress test (SKPT) and assignment of the subjects contained in the German medical licensing regulations (ÄAppO) to the subject groups.

**Table 2:**
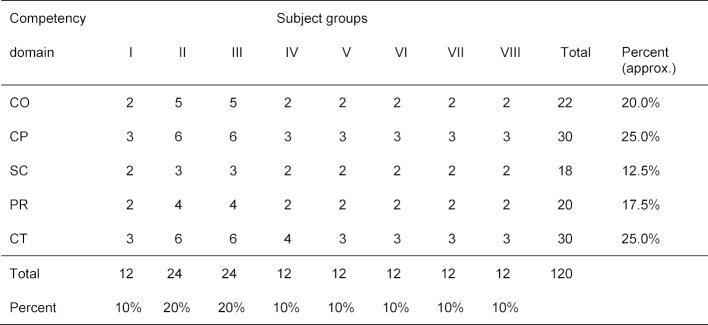
Blueprint of the competency- oriented student progress test 2014-2017 (see tab. 1 for explanation of the competency domains and subject groups).

**Table 3:**
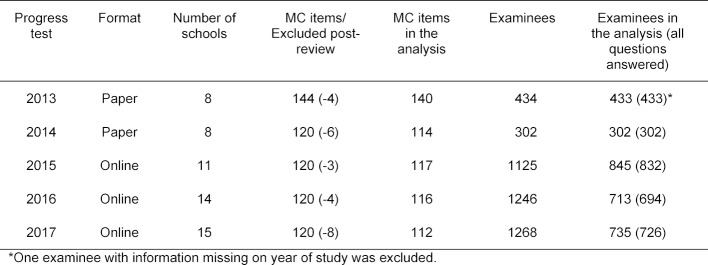
Format, number of items and number of test-takers for the SKPTs (excluding Krems medical school) in the present study. The datasets for examinees who answered at least 100 questions were included in the analysis. The number in parentheses in the last column indicates the number of examinees who answered all questions.

**Table 4:**
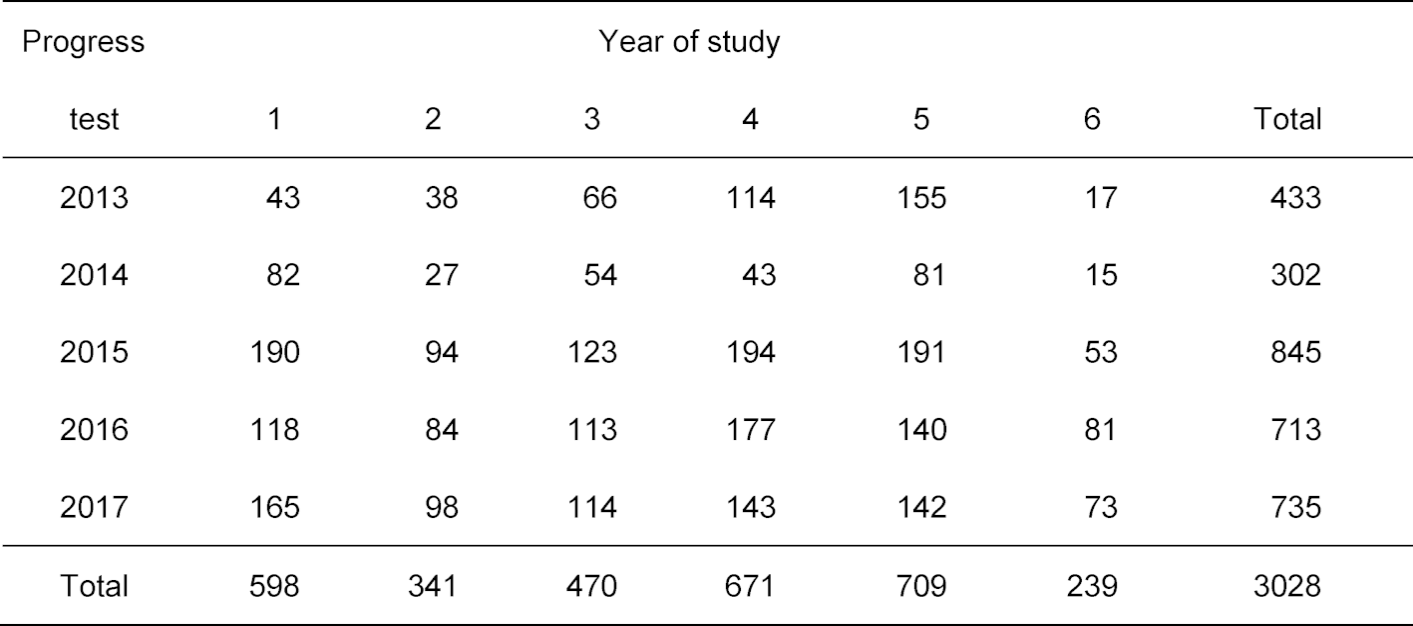
Number of participants in the analysis by year of study

**Table 5:**
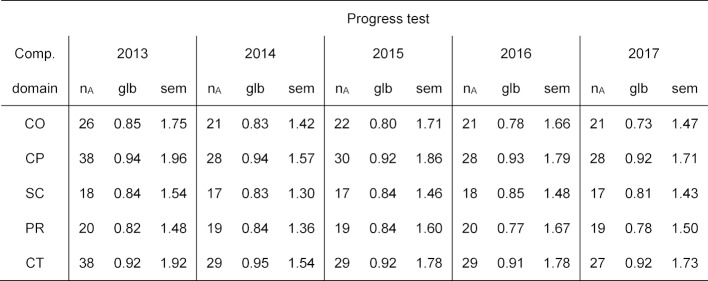
Number of questions (n_A_), reliability (coefficient glb) and standard error of measurement (sem) for the competency domains in the SKPTs for 201 –2017. Due to the exclusion of individual questions in the post-review, the numbers of questions per domain are sometimes less than intended in the blueprint (see table 2).

**Table 6:**
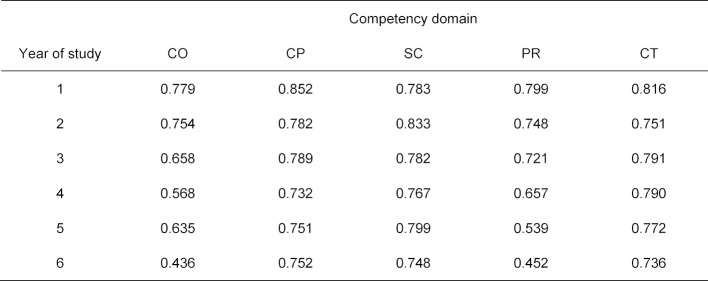
Median values of the reliabilities for each year of study and competency domain from each of the five SKPTs for 2013-2017.

**Table 7:**
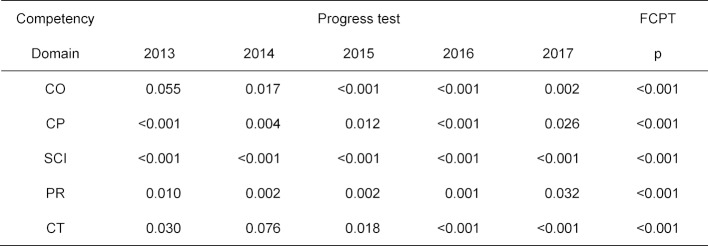
p-values for the tests for difference between the values of the discriminant function of the questions in a competency domain of the progress tests 2013-2017 in relation to the questions for all other domains and for Fisher’s combined probability test (FCPT).

**Table 8:**
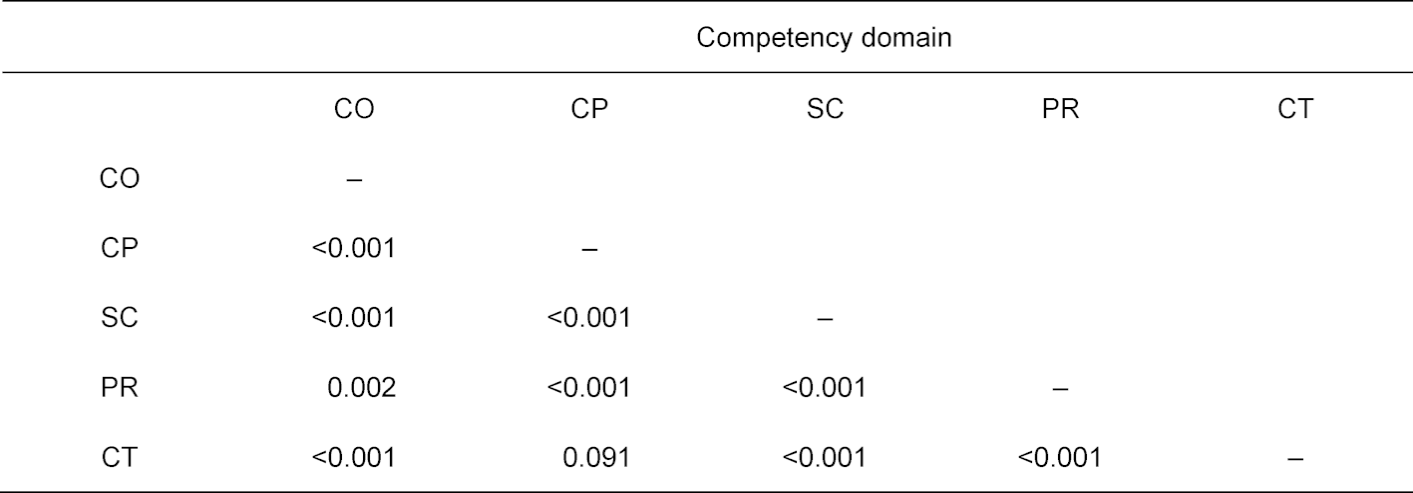
Fisher’s combined probability test (FCPT) of the pairwise tests for difference in the values of the discriminant function between the questions of two competency domains.
