# Supplementary material for: Measuring competency-relevant knowledge in the competency-oriented student progress test
Source: GMS J Med Educ. 2020 Feb 17;37(1):Doc6. doi: 10.3205/zma001299 (PMC7105759; doi:10.3205/zma001299)
Supplement: Examples of competency-specific questions from the 2017 competency-based progress test [file JME-37-6-s-001.pdf]

Examples of competency-specific questions from the 2017 competency-based progress test

The following pages contain example questions from the 2017 competency-based student progress test (SKPT) representing five competency domains:
• Communicative competence
• Practical clinical competence
• Scientific competence
• Professional decision-making competence
• Theoretical clinical competence
All of the questions from the progress tests since 2013 are available online at https://www.komp-pt.de/fragen-aus-dem-progresstest/

Communicative Competence (CO)

1 Author: Peitz, Niklas
Subject: Imaging Procedures, Radiation Therapy and Radiation Protection / Competency area: Health Advocate / Cluster 2: A / Cluster 1: I

A patient comes to your practice which also provides information on radiation protection. This patient has recently been offered a new job in which he will come into contact with radiation. His father worked at a similar job and died of lung cancer. The patient would like to accept the job offer but he is afraid that he will also get a lung tumor as a result and is asking your advice. From your perspective, he has the option to not take the job or to reduce the risk of a tumor through the use of special protective clothing and regular medical check-ups.

Which approach contributes **the least** *to a joint decision*?

(A) You discuss the different options the patient has and let him make the decision.
(B) You explain the risks to the patient and then give your recommendation.
(C) You weigh the options with him and find out how independently he wants to make this decision.
(D) You recommend not taking the job to the patient so that he is not exposed to any psychological stress.
(E) You ask if the job is attractive to him independent of the risks and, if so, you then try to make the job possible for him.
(F) I don't know.

Reference:
Bieber et Al.: Partizipative Entscheidungsfindung (PEF) - Arzt und Patient als Team (see Google Drive)

Practical Clinical Competence (CP)

4 Author: Jin, Jia xiang

Subject: Imaging Procedures, Radiation Therapy, Radiation Protection / Competency area: Application of diagnostic procedures / Cluster 2: B / Cluster 1: I

You are working at a general medical practice which offers mammograms as a diagnostic procedure for early detection of breast cancer.

For which of the following patient profiles would you be most likely to recommend regular screening for the purpose of early detection?

(A) Mrs. Mueller: age 45, no known family history of breast cancer; she desires screening because someone among her friends has recently died of breast cancer.
(B) Mr. Mueller: age 46, breast cancer was diagnosed in a maternal aunt; he is accompanying his wife.
(C) Ms. Koch: age 22, the Mueller's daughter.
(D) Mrs. Mueller-Schmidt: age 65, mother of Mr. Mueller, just recently she has felt something hard in her breast.
(E) Mr. Mueller: age 71, husband of Mrs. Mueller-Schmidt and father of Mrs. Mueller.
(F) I don't know.

Reference:
https://www.uspreventiveservicestaskforce.org/Page/Document/UpdateSummaryFinal/breast-cancer-screening1?ds=1&s=breast%20cancer

Scientific Competence (SC)

26 Author: Peitz, Niklas

Subject: Infectious Disease/Immunology / Competency area: Scholar / Cluster 2: C / Cluster 1: II

You are planning to serve your clerkship abroad and therefore wish to inform yourself about tuberculosis in India and China. Tuberculosis (TB) is endemic in both countries. You find the following statistics for the year 2015:

Which statement can you make based solely on the table?

India

Population 2015 **1 311 million**

Estimates of TB burden*, 2015	Rate (per 100 000 population)
Mortality (HIV+TB only)	2.8 (1.6–4.3)
Incidence (includes HIV+TB)	217 (112–355)

China

Population 2015 **1 376 million**

Estimates of TB burden*, 2015	Rate (per 100 000 population)
Mortality (HIV+TB only)	0.19 (0.09–0.33)
Incidence (includes HIV+TB)	67 (57–77)

(A) In India there are relatively more people who have newly contracted TB.
(B) TB progresses more rapidly and severely in India.
(C) In China the chances of surviving TB are lower than in India.
(D) The absolute number of people with TB is higher in China.
(E) In India the healthcare system is overwhelmed by TB infections.
(F) I don’t know.

Reference:
https://extranet.who.int/sree/Reports?op=Replet&name=%2FWHO_HQ_Reports%2FG2%2FPROD%2FEXT%2FTBCountryProfile&ISO2=IN&LAN=EN&outtype=pdf
https://extranet.who.int/sree/Reports?op=Replet&name=%2FWHO_HQ_Reports%2FG2%2FPROD%2FEXT%2FTBCountryProfile&ISO2=CN&LAN=EN&outtype=pdf

Professional Decision Making Competence (PR)

52 Author: Müller, Andreas
Subject: Surgery / Competency area: Ethics and Law / Cluster 2: D / Cluster 1: III

You are working as an assistant physician on an orthopedic and emergency surgery ward. An 87-year-old female patient has been given a duo head prosthesis following a medial hip fracture. This replaces the femur head while preserving the acetabulum. The patient is receiving physiotherapy, but has not made any noticeable progress in the week since the operation: the patient can at most be mobilized to the edge of the bed. The patient is somewhat forgetful, yet well oriented and not limited in her ability to communicate. She is polypharmacized with many medications for hypertension, diabetes and other internal ailments. You are considering if the patient should apply for rehabilitation ("rehab").

Which factor most likely reduces the chances of a successful application for rehab?

(A) The acetabulum-preserving endoprothesis.
(B) The minimal progress in improving mobility.
(C) The neuropsychological status of the patient.
(D) Medication with multiple drugs.
(E) I don't know.

Reference: http://www.deutscherentenversicherung.de/cae/servlet/contentblob/208282/publicationFile/2266/ahb_indikationskatalog.pdf , Page 3

Theoretical Clinical Competence (CT)

31 Author: Grupp, Margarethe
Subject: Microbiology, Virology, Hygiene / Competency area: Pathophysiological mechanisms / Cluster 2: E / Cluster 1: II

A 43-year-old female patient appears at your general medical practice. She presents with fever, chills and is generally fatigued. She is also short of breath and coughing up yellow-green sputum. These symptoms have appeared very suddenly. There is no history of disease. During the physical exam you find the following:
Temperature: 38.9°C
Blood pressure: 110/65 mmHg
Breathing rate: 26/min
Increased fremitus
Percussory dull sound in the lower right lobe.
Asculation reveals fine crackles in the lower right lobe.
Lab results: leukocytosis; CRP 80 mg/l; procalcitonin 0.46 ng/ml.
Which is the most probably pathogen in this case?

(A) Haemophilus influenzae
(B) Mycoplasma pneumoniae
(C) Chlamydia pneumoniae
(D) Pseudomonas aeruginosa
(E) Streptococcus pneumoniae
(F) I don't know.

Reference: http://www.awmf.org/uploads/tx_szleitlinien/020-020l_S3_ambulant_erworbene_Pneumonie_Behandlung_Praevention_2016-02-2.pdf
